# An Automated Continuous Synthesis and Isolation for the Scalable Production of Aryl Sulfonyl Chlorides

**DOI:** 10.3390/molecules28104213

**Published:** 2023-05-20

**Authors:** Matthew Glace, Cameron Armstrong, Nathan Puryear, Colin Bailey, Roudabeh Sadat Moazeni-Pourasil, Drew Scott, Sherif Abdelwahed, Thomas. D. Roper

**Affiliations:** 1 Department of Chemical and Life Sciences Engineering, Virginia Commonwealth University, Richmond, VA 23284, USA; glacemk@vcu.edu (M.G.); armstrongct2@vcu.edu (C.A.); baileycd2@vcu.edu (C.B.); moazenipourr@vcu.edu (R.S.M.-P.); scottma3@vcu.edu (D.S.); 2 Department of Electrical and Computer Engineering, Virginia Commonwealth University, Richmond, VA 23284, USA; puryearna@vcu.edu (N.P.); sabdelwahed@vcu.edu (S.A.)

**Keywords:** flow chemistry, chlorosulfonation, process automation, pump control system, continuous manufacturing, batch design

## Abstract

In this work, a continuous system to produce multi-hundred-gram quantities of aryl sulfonyl chlorides is described. The scheme employs multiple continuous stirred-tank reactors (CSTRs) and a continuous filtration system and incorporates an automated process control scheme. The experimental process outlined is intended to safely produce the desired sulfonyl chloride at laboratory scale. Suitable reaction conditions were first determined using a batch-chemistry design of experiments (DOE) and several isolation methods. The hazards and incompatibilities of the heated chlorosulfonic acid reaction mixture were addressed by careful equipment selection, process monitoring, and automation. The approximations of the CSTR fill levels and pumping performance were measured by real-time data from gravimetric balances, ultimately leading to the incorporation of feedback controllers. The introduction of process automation demonstrated in this work resulted in significant improvements in process setpoint consistency, reliability, and spacetime yield, as demonstrated in medium- and large-scale continuous manufacturing runs.

## 1. Introduction

Chlorosulfonates are precursors to a wide range of biologically active molecules, including sulfonylureas, sulfonamides, and many sulfinate derivatives [1,2,3,4,5,6]. These drug classes have properties that include antimicrobial, anti-tumor, and diuretic effects [3,6,7]. Numerous active pharmaceutical ingredients (APIs) that have been designated essential medicines by the World Health Organization rely on chlorosulfonation chemistry during production [8]. An uncomprehensive list of some important commercial drugs relying on sulfonyl functionalization is shown in Table 1. While the global consumption for many of these drugs is not readily available, sildenafil citrate alone had a monthly consumption of around 5 million units in 2020 and has been a Pfizer product since 1998 [9,10]. 

Significant efforts have been directed towards the development of ‘green’ processes for chlorosulfonation chemistry [18,19,20]. Chlorosulfonation generally requires harsh reaction conditions and a high excess of reagents for industrial manufacturing [21]. For these reasons and others, chlorosulfonation reactions are good potential candidates for flow chemistry implementation. Flow chemistry is being employed to improve the safety, speed, product quality, and environmental footprint of challenging reactions [22,23,24]. Broadly, the utilization of flow chemistry for API manufacturing is increasing significantly [25,26,27,28,29,30,31,32,33,34,35,36], as encouraged by regulatory bodies [37,38]. Previous works have utilized flow chemistry to address the environmental and safety challenges associated with chlorosulfonation reactions [21,39,40]. While these studies have demonstrated the advantages of flow chemistry, their production scale has not been shown to be feasible for commercial manufacturing. 

Chlorosulfonic acid (ClSO_3_H) is a popular reagent to produce chlorosulfonates due to its ability to selectively chlorosulfonate aryl rings via an S_E_2 mechanism [41]. In recent years, chlorosulfonic acid has been reported in the synthesis of sildenafil [12], antibacterial quinoxaline [4], sulfonamides, antineoplastic sulfonamides [7], and an anti-tumor drug [42]. Despite the prolific use of chlorosulfonic acid, flow chemistry methods that employ ClSO_3_H have only rarely been described [21,43]. Furthermore, significant technical challenges are introduced as the reaction is scaled to commercial feasibility. Particularly, the reliable handling of heated, highly corrosive ClSO_3_H was critical for its successful development. 

The chlorosulfonation reaction reported was the first step from commercially available starting material for a confidential drug target in development at the Medicines for All Institute at Virginia Commonwealth University. In this case, a continuous manufacturing process for the use of ClSO_3_H was developed as a viable alternative to a batch process that was otherwise considered too hazardous for scale-up in our facilities. The reaction conditions were first optimized using design of experiments (DOE), which has previously proven to be a common and valuable tool for pharmaceutical optimization [44,45,46,47]. Intermediate, scaled-up batch experiments were next performed to address the challenges of the highly exothermic quenching procedure. Finally, the translation from batch experiments to a 500 g flow-capable process with level monitoring and feedback control is delineated. Hardware and software obstacles were addressed in a collaborative effort between pharmaceutical development and system control research groups. The implemented process is beneficial to improving the safety and spacetime yield of a highly corrosive reagent. 

## 2. Materials and Methods

### 2.1. Chemicals

All materials were used as received from vendors. ClSO_3_H (99%), diethylene glycol dimethyl ether (99%, Diglyme), and acetonitrile (99%) were procured from Sigma Aldrich (St. Louis, MO). The unspecified starting material (>98%) was procured from TCI Chemicals (Tokyo, Japan). Silicon heating oil was procured from Beantown Chemical (Hudson, NH, USA).

### 2.2. Analytical Procedure

Samples were analyzed via high-performance liquid chromatography (HPLC) with ultraviolet detection, as described in Section 5.1 of the supporting information. Depending on the amount of solid product, between 1 and 10 samples were averaged per experiment. For area and weight percent analyses, samples were prepared in duplicate at ~1 mg/mL in acetonitrile and analyzed in duplicate (n = 4). Duplicate standards were analyzed throughout the sequence.

### 2.3. Software

Preliminary process monitoring was performed in Python via a serial port connection to weighing scales. Node-RED [48,49] was used as the developing environment for the proportional–integral–derivative (PID) process controller. 

### 2.4. Batch Chemistry: Screening Scale

A silicon oil bath was pre-heated to experimental temperature. In a 25 mL two-neck round-bottom flask (RBF), 5.0 g of starting material was added. A magnetic stir bar and the molar equivalents of ClSO_3_H were added. The RBF was added under a reflux condenser with nitrogen purge and a glass stopper, lubricated with grease. The RBF was submerged in the preheated oil bath, marking the reaction start time. Timepoint samples were prepared for HPLC at 8 time points (5, 10, 15, 20, 30, 60, 90, and 120 min) by quenching several drops of the cooled reaction mixture in chilled water (0–5 °C). The slurry was diluted with acetonitrile to dissolve it to approximately 1 mg/mL.

### 2.5. Batch Chemistry: Scale-up Procedure

In a 250 mL 3-neck round-bottom flask, 8 equivalents of chlorosulfonic acid was chilled to −25°C. The substrate (50 g) was added portion-wise over 20 min, maintaining temperature between −20 °C and −25 °C. Lubrication grease was applied to the reflux condenser base, nitrogen line adapter, Teflon J-Kem adapter, and glass stopper to prevent fumes from leaking from the RBF. After 10 min of stirring, the funnel was replaced, the reflux condenser was under nitrogen with a base trap, and taper clips were added to all components. The reaction was heated to reflux in a silicone oil bath, and internal temperature was maintained between 151 °C and 156 °C. An in-process HPLC sample was taken by quenching in water and then dissolving in ACN to verify reaction completion. The reaction mixture was cooled to room temperature and pumped into a European 3-neck jacketed 1 L vessel with 400 mL H_2_O and 40 mL Diglyme set to −10 °C. A mechanical overhead stirrer was used at 200 RPM through the center port. A temperature probe and nitrogen inlet line were inserted through a sealed adapter at the second neck of the flask, with the third port being a gaseous outlet into a base trap of concentrated NaOH. The addition rate to the quenching was 2 mL/min over approximately 2 h. An internal temperature probe was added to the quenching mixture to verify that the temperature stayed between −2 °C and −8 °C. After 10 min of stirring (post quench completion), the product slurry was added into a sinter funnel, filtered under vacuum, washed with cold water, and dried in a vacuum oven with a nitrogen bleed at 40 °C. The product was verified by 1H-NMR, HPLC Mass Assay, and LCMS.

### 2.6. Flow Chemistry: Procedure

**Figure 1:**
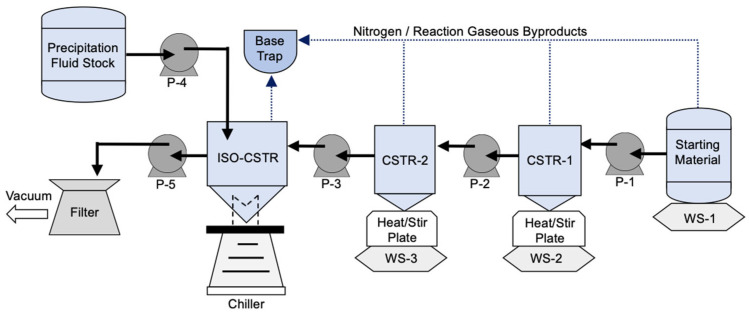
Process Flow Diagram.

The finalized process flow diagram for continuous chlorosulfonation and isolation is shown in Figure 1. Peristaltic pumps (P-) were employed for material transfer between two consecutive continuous stirred-tank reactors (CSTR-1,2), followed by continuous stirred-tank isolation (ISO-CSTR) and filtration of the slurry. Mettler Toledo weighing scales (WS) were placed under the starting material stock and CSTR-1/2 for gravimetric measurement.

For each CSTR, borosilicate round-bottom flasks (50 or 500 mL) with magnetic stir bars were heated in a silicon oil bath by an IKA (Staufen, Germany) RCT digital heating/stirring plate and measured by Mettler Toledo (Columbus, OH, USA) weighing scales. Round-bottom flasks were either placed under water-cooled reflux condensers or Heidolph (Wood Dale, IL, USA) Findensers. Both setups were headed with nitrogen through a gas manifold that was bubbled into a base trap of concentrated sodium hydroxide to help reduce acidic fumes. Precipitation was either performed in a jacketed 1000 mL 3-neck European flask (Chemglass, Vineland, NJ, USA) or 20 L jacketed batch reactor (Chemglass, Vineland, NJ, USA), both attached to a Julabo (Seelbach, Germany) chiller. Cole-Parmer (Vernon, IL, USA) Masterflex peristaltic pumps (Model No. 07522-28) were used either with C-Flex or PTFE pump heads. C-Flex tubing sizes 13, 14, and 15 and PTFE tubing (1/8′ OD, 1/16′ ID) were used in conjunction for early experiments. For latter runs, a PTFE tube set (2 mm ID, 4 mm OD) from Masterflex was connected to PTFE tubing (1/8′ OD, 1/16′ ID) with Idex P-330X Flangeless Male Nuts, locking ferrules, and PEEK unions.

In a Pyrex bottle, ClSO_3_H was charged with the starting material at −20 °C and then warmed to room temperature under nitrogen. Stability testing was carried out to ensure no reactions or degradation occurred at room temperature during the length of the experiment. For startup, each CSTR was filled with reaction mixture and held for 2 h at reflux (~155 °C) before pumping began to maintain 1 h in each CSTR. The ISO-CSTR was initially filled with chilled blank precipitate fluid. The product from CSTR-2 was added to the ISO-CSTR mixture along with the antisolvent mixture. The peristaltic pumps used for continuous precipitation and filtration were set manually during all experiments. Intermittent periods of high flowrate were utilized to obtain the desired time-averaged outlet flow from ISO-CSTR, which is further detailed in supporting information Table S1. For shutdown, the reaction CSTRs were drained into the ISO-CSTR at the same setpoint flowrate. The ISO-CSTR was drained into the filter and washed with cold water to collect all the solid product. The solid was washed with cold H_2_O and dried in a vacuum oven with a slow nitrogen bleed at temperatures no higher than 50 °C. 

### 2.7. Flow Process: Monitoring/Control

The first two-hour period of the startup program was automated to resemble a batch process. In summary, both CSTRs were filled with reaction mixture and held at the reaction conditions before beginning steady-state operation. To achieve this, P-1 was first set to rapidly fill CSTR-1 to the targeted volume. Once WS-2 indicated the targeted volume had been achieved, P-1 reduced speed by 10%, and P-2 began to rapidly fill CSTR-2. The 10% difference in speed was to prevent CSTR-1 from overfilling in the instance of unequal pumping performance between P-1 and P-2. Once WS-3 indicated CSTR-2 had filled, P-2 stopped and P-1 continued to top off CSTR-1. After both CSTR-1 and CSTR-2 achieved target volumes, the reaction was held for two hours before beginning steady-state operation. 

The industrial standard startup of filling both CSTRs with ClSO_3_H followed by the steady-state addition rate of reaction stock did not seem suitable for a laboratory setting for several reasons. This procedure would increase the amount of ClSO_3_H necessary to complete the experiment and the total amount of waste produced. Given the limited availability of the starting material, it was critical to collect as much of the sulfonyl chloride product as possible. Batch data showed that overreaction was not problematic. Lastly, the increased rate of off-gassing products in CSTR-1 and CSTR-2 from the batch method startup was less concerning than the fumes produced during the precipitation in ISO-CSTR. Notably, P-3 was programmed so that the highest voltage input signal possible from the Raspberry Pi still corresponded with a safe flowrate into the precipitation of around 4 mL/min. For instances of scale-up where venting of CSTR-1 and CSTR-2 must be considered beyond a gas manifold with a nitrogen purge to a base trap, this startup method may be unsuitable. 

For steady-state operation, the first PID controller ensured an accurate flowrate of the feedstock into CSTR-1. This was achieved by adjusting the rotational speed of P-1 to hold the time derivative of WS-1 at the desired setpoint. The second PID controller utilized mass data from WS-2 and WS-3 to maintain the desired fill level in CSTR-1 and CSTR-2 by adjusting P-2 and P-3. Both PID controllers working together ensured a consistent and accurate flowrate of P-1, P-2, and P-3 as well as the CSTR fill levels to tightly control the residence time. Parameters for each of the PID controllers were determined empirically. The specific PID controller implementation, provided by a third-party library, utilized untraditional parameter specifications. Instead of specifying the gain values for the proportional, integral, and derivative terms directly, these values were determined based on the parameters available with this specific controller implementation. Based on the empirically determined parameters used after finalizing the tuning, the controllers were essentially PI controllers, where the output was mainly determined by the proportional term.

**Figure 2:**
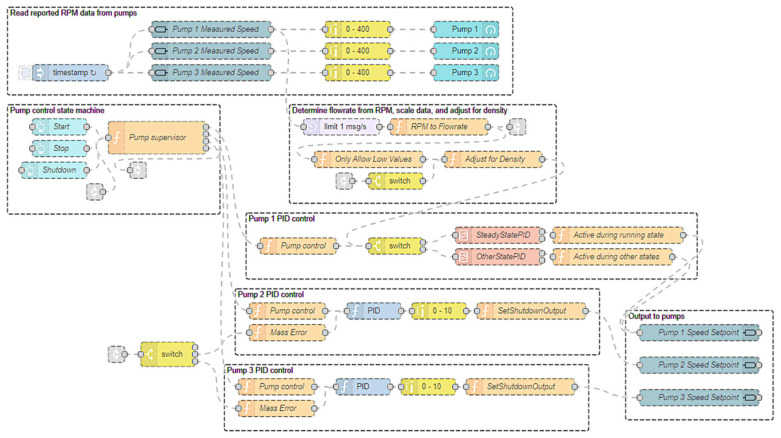
Main Node-RED Program Flow.

In order to develop a PID-based control system, Node-RED was selected and installed on the Raspberry Pi to provide a web-based development environment. Node-RED is an intuitive and powerful NodeJS-based programming environment that utilizes program flows to develop the software to control the attached hardware. Programming nodes are placed and wired together to provide an event-based application. In addition to the default nodes, other third-party libraries were installed, which provided nodes for controlling the industrial automation interface board, nodes for developing a web-based dashboard, and a PID controller node. Multiple Node-RED flows were created for this application, with the main flow shown in Figure 2. 

At the top, the first group of nodes is responsible for reading the 0–10 V analog outputs from the pumps and converting them to an appropriate RPM value. These nodes were mainly used on the dashboard to ensure the pumps were actively running. Just below this group is another group of nodes that implement a state machine for the system. User input was taken from the dashboard to start, stop, and shutdown the process. The “Pump supervisor” node contains a JavaScript function that takes the user’s input as well as the scale data to determine the current state of the system. This state information is then passed to the PID controllers for each of the three pumps. 

For P-2 and P-3, the PID controllers are identical and operate by reducing the error between the mass values reported by the scales and a desired setpoint. These two PID controllers only operate during the steady-state operation of the system. During the startup and shutdown states, the outputs of the second and third pumps are held at a constant value as determined by the pump supervisor node. Each PID controller operates by setting its output as a percentage of the total output (a value between 0 and 1). This output value is determined by the difference between the current value of the system and the setpoint (the proportional term), the accumulated error over time (the integral term), and the instantaneous change in error (the derivative term). The output is then scaled to the required 0–10 V output for the pumps and output using the interface board’s output nodes. These PID controllers for the second and third pumps allow the desired mass to be held almost constant, while P-1 delivers starting material into the system at a constant rate as dictated by a separate PID controller. This effectively results in P-1 controlling the flowrates for P-2 and P-3 during steady-state operation.

**Figure 3:**
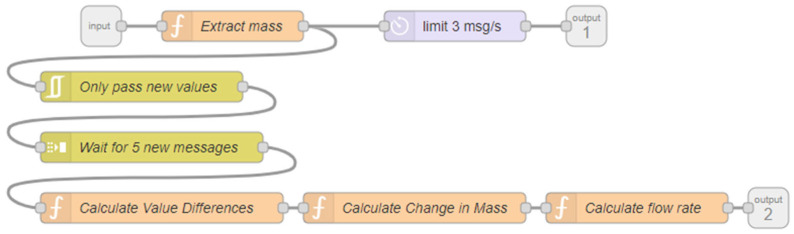
Scale Data Processing Node-RED Flow.

While P-2 and P-3 directly used the mass reported by the WS-2 and WS-3 as their inputs, a different solution was required to implement the flowrate-based PID controller for P-1. The flowrate from the stock solution needed to be calculated via the change in mass from WS-1. The Mettler Toledo scales used in this system provided updated mass values approximately 20 times per second; however, because the precision of the scales was 0.1 g, calculating the flowrate using the change in mass over time from every sample value resulted in periods of a flowrate of 0 reported, followed by sporadic spikes in flowrate values when the mass value changed. To mitigate this, the Node-RED flow in Figure 3 was added.

In this flow, the mass reported by the scale was still provided as one output (limited to 3 messages per second to improve computational performance), but another output for the flowrate was added. To determine the flowrate, only changes in mass were passed through the flow. Then, a buffer of five messages was filled, from which the first and fifth messages were used to calculate the mass flowrate. This allowed for a more accurate representation of the average flowrate as the mass flowrate was calculated over a longer period of time. The mass flowrate was then converted to a volumetric flowrate using the density of the solution before being sent to the first pump’s PID controller, as shown in Figure 2.

Because of the filtering steps added in calculating the mass flowrate, the frequency at which the flowrate was calculated was directly proportional to the speed of the pump. Therefore, when the pump was at very low rotations per minute (RPM), the flowrate could take several seconds, or even minutes, to update. This was problematic because the PID controller node only updates the output control value when the input value changes. To solve this issue, nodes were added to use the reported RPM value from P-1 when estimating the flowrate when the RPM was very low. An empirically determined constant value was multiplied by the pump’s reported RPM value to approximate the mass flowrate. These approximated values were only allowed as inputs to the PID controller when they were less than 1 mL/min.

## 3. Results and Discussion

### 3.1. Batch Chemistry Design of Experiment

**Figure 4:**
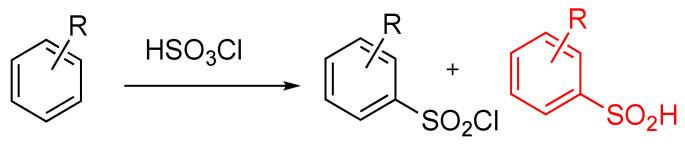
General Scheme for Chlorosulfonation.

The scheme for the chlorosulfonation reaction is outlined in Figure 4. Both the desired sulfonyl chloride (product) and the undesired sulfonic acid (impurity) are shown. The sulfonic acid can be generated both during the reaction as an intermediate and as an impurity during the isolation. A DOE was first performed utilizing the batch procedure delineated in Section 2.4. The objective outcome of the batch development DOE was the selection of experimental conditions to maximize the yield of the sulfonyl chloride and minimize the starting material. Additionally, the collection of reaction timepoints was intended to aid in the kinetic model for CSTR sizing. The central composite design (CCD) method was the primary response surface methodology used to characterize the batch experiments. The preliminary experiments suggested that the most critical parameters were temperature, ClSO_3_H equivalents, and time. For each reaction condition, the concentration of the starting material, the desired sulfonyl chloride product, and the sulfonic acid (impurity) were measured using HPLC analysis at several timepoints. The process parameters and the desired goals are summarized in Table 2. The experimental list was designed by a rotatable CCD and is shown in Table 3.

**Figure 5:**
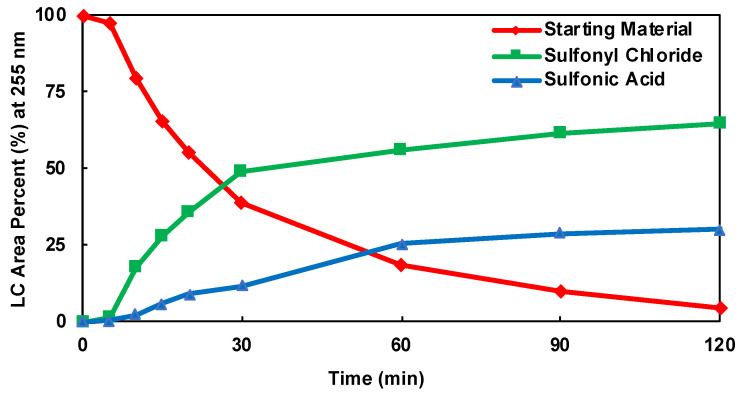
Kinetic Plot of DOE Batch Experiment 2.

**Figure 6:**
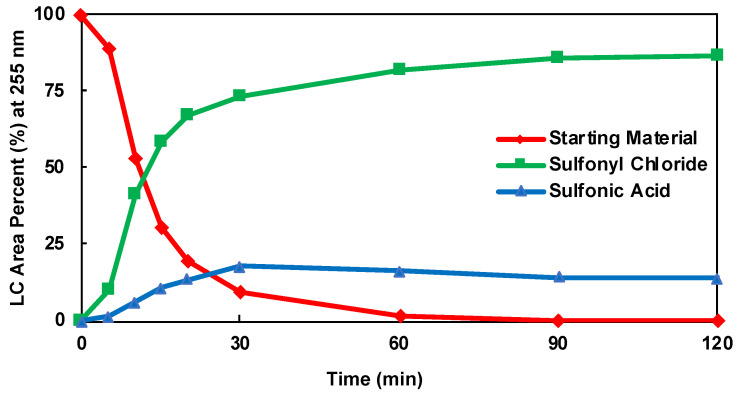
Kinetic Plot DOE Batch Experiment 3.

**Figure 7:**
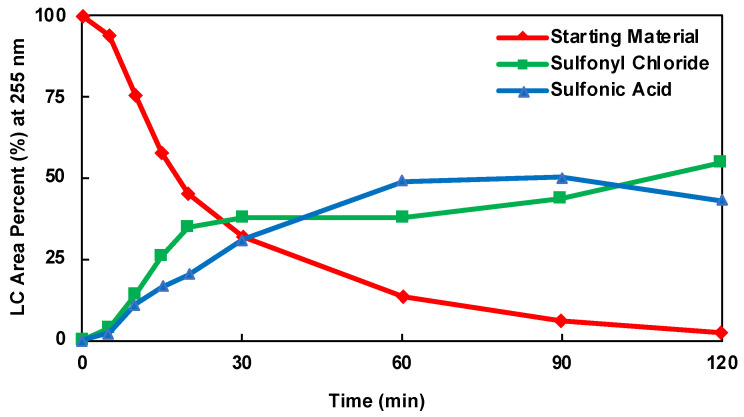
Kinetic Plot of DOE Batch Experiment 10.

The HPLC area percent (LCAP) of the product, sulfonic acid, and starting material for each DOE batch experiment was plotted as a function of time. The kinetic plots for DOE batch experiments 2, 3, and 10 are shown in Figure 5, Figure 6 and Figure 7, respectively. The kinetic plots for the remaining DOE batch experiments can be found in the supporting information (Figures S3–S10). Across all 11 experimental conditions, the reaction appears to be near equilibrium around 60 min. Therefore, the 60 min HPLC data were considered the basis for statistical data analysis and optimization. An ANOVA analysis was performed for parameter fitting and significance determination in the models of the product, starting material, and sulfonic acid. The ANOVA tables for each model are shown in supporting information Tables S2–S4. For each term in the model, a *p*-value of less than 0.0500 was considered significant and included. A cubic model was fitted for the product (R1), a quadratic model for the starting material (R2), and a quadratic model for the inverse of sulfonic acid (R3). The responses for each model, including the significant factors, are shown in Equations (1)–(3).

R1 = 58.02 + 14.24 A + 8.13 B −3.64 AB +2.84 A^2^ − 4.55 B^2^ − 7.92 A^2^B − 0.7728 AB^2^ (1)

R2 = 14.81 − 16.22 A + 2.18 B + 1.45 AB + 4.84 A^2^ + 2.03 B^2^ (2)

1/R3 = 0.0376 − 0.0101 A + 0.0108 B − 0.0104 AB + 0.0168 A^2^ (3)

**Figure 8:**
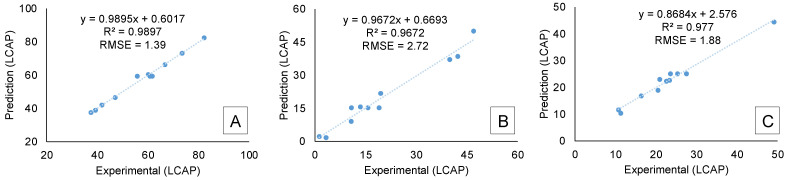
Fit between DOE Simulation and Experimental Results at the 60 min timepoint for (**A**) Sulfonyl Chloride Product, (**B**) Aryl Starting Material, and (**C**) Sulfonic Acid Impurity.

The fitting for all the DOE batch experiments at the 60 min timepoint was compared with the theoretical DOE model. The results for the product, starting material, and sulfonic acid are shown in Figure 8A–C. Both the correlation coefficients (R^2^) and root mean square errors (RMSE) indicate a good fit between the theoretical DOE model and the experimental results.

Multivariate optimization was next conducted by introducing a desirability function. The criteria applied for multivariate optimization were to find the temperature and ClSO_3_H equivalents in the range that maximized R1, minimized R2, and kept R3 within the range of the batch experiments. A solution with 100% desirability was selected and is shown in Table 4. Although the combined LCAP of the three components is 104.9 rather than 100.0, this is merely an effect of each component being modeled individually. The optimized condition should serve as a benchmark for reaction yield and purity for the crude reaction mixture, specifically the product LCAP. 

### 3.2. Batch Chemistry Scale-Up

As an intermediate scale-up (SU) prior to the implementation of the continuous process, batch experiments were conducted at a 50 g starting material basis, as outlined in Section 2.5. The results are summarized in Table 5. Because batch chemistry development and DOE were performed in parallel to the implementation of the continuous process, the SU-1 conditions were reflected in the first two unoptimized continuous experiments. The optimized conditions adapted for the latter flow experiments (3 and 4) are reported in SU-2. These experiments served as benchmarks of yield and purity for the isolated solid from the continuous process. 

Several adaptations were required to address the scalability and environmental metrics of the highly exothermic quench and solid recovery. The procedure changes reflect the objectives of increasing isolated yield, improving safety, and reducing the solvent volume required for the quench. Following the results of the preliminary batch screening, the equivalents of ClSO_3_H were increased, which improved the isolated yield from 67.3 to 87.7%. Utilizing an organic co-solvent for the precipitation lowered the freezing point of the precipitate fluid and reduced the amount of water required to isolate the solid sulfonyl chloride by 80%. In addition, the inclusion of diglyme resulted in a powder product with qualitatively higher flowability. Green chemistry metrics [50] were improved throughout chemistry development, and the E-factor was also reduced nearly sevenfold from 80 (SU-1) to 12 (SU-2). 

As the process was scaled to half-kilogram production, the chlorosulfonation reaction and isolation became critical points for material throughput due to safety concerns and physical process limitations. A risk assessment of the scale-up revealed it was unsafe to rapidly heat a large volume of crude reaction mixture as loss of containment for the heated ClSO_3_H was considered too dangerous for personnel and facilities. Additionally, a large amount of gas was released first during the reaction and more so during the isolation. Although a gas scrubber was utilized for both the reaction and precipitation, it was difficult to maintain a consistent rate of off-gassing for the batch process, which led to generally hazardous operating conditions.

For the batch process, the possible spacetime yield of sulfonyl chloride product was limited both by the time required for the reaction mixture to reach reflux and by the necessarily slow addition to the precipitation. Kinetics dictate that the reaction be completed within two hours, but for ‘batch’ chemistry, the total time to isolate the solid product was over three times that. Increasing the rate of addition into the highly exothermic precipitation resulted in reduced yields, higher levels of the sulfonic acid impurity, and increased trapped water content of the sulfonyl chloride product that could not be readily removed. Safety and throughput restrictions were not easily addressed for the batch process outlined. Considering such limitations, it was decidedly advantageous to develop a general continuous manufacturing platform for both chlorosulfonation and isolation.

### 3.3. Flow Chemistry Design

While the chlorosulfonation process was briefly attempted in a small-scale plug flow reactor (PFR), it became readily apparent that it was unsuitable for this application. The accumulation of gaseous byproducts was problematic for the residence time distribution. The pressurization required for a PFR was identified as a safety risk for a superheated, highly corrosive solvent. Additionally, chemically compatible hardware options for pumping were further limited by the pressure requirement. Compared to the batch process, the application of a PFR seemed to introduce more problems than it solved. To perform the reaction in flow, a series of continuous stirred-tank reactors (CSTRs) was chosen. The utilization of CSTRs facilitated the removal of gaseous byproducts with an inert purge in gas scrubbers and the use of peristaltic pumps with no wetted mechanical components. The reaction vessels remained unpressurized and operated under reflux conditions.

(4) $$\frac{C_{AF}}{C_{A0}}=e^{-kt}$$

Kinetics were based on DOE batch experiment 3 (Figure 6), which was close to the multivariate optimal solution. With the high excess of ClSO_3_H used, the reaction was assumed to be pseudo-first order with respect to the starting material, as shown in Equation (4). The starting material concentration and final concentration are represented as *C*_*A*0_ and *C_AF_*, respectively. In-process HPLC monitoring showed nearly 99% starting-material conversion after 60 min. For a 60 min reaction time at 99% conversion, a first-order approximation gives a rate constant *k* ≈ 0.077 min^−1^.

(5) $$\frac{C_{AF}}{C_{A0}}={\left(\frac{1}{1+k\tau }\right)}^{n}$$

For *k* = 0.077 min^−1^, 97% conversion of starting material was estimated in two CSTRs (*n* = 2), with each residence time (τ) being equal to 60 min (Equation (5)). The implementation of CSTRs in series also significantly accelerated the heat transfer compared to the batch process, which nearly eliminated the extra time required to bring the starting material up to reflux temperature. Based on the model results, the utilization of two CSTRs in series was advantageous in reducing the heated ClSO_3_H necessary to achieve the desired process throughput. The reduction in the heated ClSO_3_H was considered a major process safety improvement that facilitated further reaction scale-up. 

### 3.4. Flow Chemistry and Process Control

A continuous manufacturing chlorosulfonation reaction and precipitation were designed and performed utilizing the procedure and configuration outlined in Section 2.6 and Figure 1, respectively. The flow chemistry conditions were selected with the objectives of safely improving spacetime yield and environmental metrics and demonstrating the batch DOE model design. Another objective of the continuous scheme was the development of a robust process capable of accurately maintaining the desired mean residence time. The optimization of the chemistry conditions and isolation parameters and the development of an effective process control strategy were demonstrated over four continuous manufacturing experiments. The changes to chemistry, tubing, monitoring, and control for the continuous manufacturing experiments are summarized in Table 6. 

Throughout the four continuous manufacturing runs, new layers of process monitoring and control were progressively implemented (Section 2.7). Direct monitoring of flowrates was not possible as ClSO_3_H would corrode most common flow sensors. Weighing scales (WS) were applied to monitor feedstock and reactor volumes, enabling flowrate calculations. Initially, weighing scales were connected to a Python terminal via a serial port connection, and masses were exported to a CSV file once every minute. The mass was converted into volume using a measured density and assumed to be constant as the reaction progressed. Although the weighing scales were useful for process monitoring, it was too difficult to manually adjust the process parameters in real time quickly enough to make useful changes, leading to the final employment of process controllers. PID controllers were implemented during Run 4. The application of PTFE pump heads was a prerequisite to the deployment of PID controllers that utilize scale data because tubing changes or process interference of any kind would have altered the mass reading of the balance and resulted in an inaccurate fill volume. Masterflex peristaltic pumps were controlled via a Raspberry Pi 3 Model B+ with an industrial automation stackable card to DB25 pinouts. The Mettler Toledo scales were also connected to the Raspberry Pi via USB. The versatility of the PID controller allowed for fully automated start-up, steady state, and shutdown sequences. 

Following the suggestions from the batch DOE model, ClSO_3_H was raised from 6 to 8 equivalents. For the isolation, reduction below 40 volumes of water resulted in a hard, clumpy product with high residual water content and reduced purity. Following the intermediate batch scale-up results (Table 5), diglyme was introduced in Run 3 to lower the freezing point of the precipitate fluid and provide a product with higher flowability.

For the first continuous manufacturing experiment, only WS-1 was used to monitor the remaining mass of the feedstock solution. A consistent average flowrate (0.502 mL/min, R^2^ = 0.9952) at or near the desired setpoint (0.5 mL/min) was maintained throughout the duration of the experiment (Figure S11). Pump 1 (P-1), which transferred the feedstock to CSTR-1, was less problematic than P-2 and P-3 as the feedstock was not heated. Visual inspection of the CSTR levels throughout the duration of the experiment revealed a need to implement the additional weighing scales WS-2 and WS-3 under CSTR-1 and CSTR-2, respectively, to approximate the fill levels.

**Figure 9:**
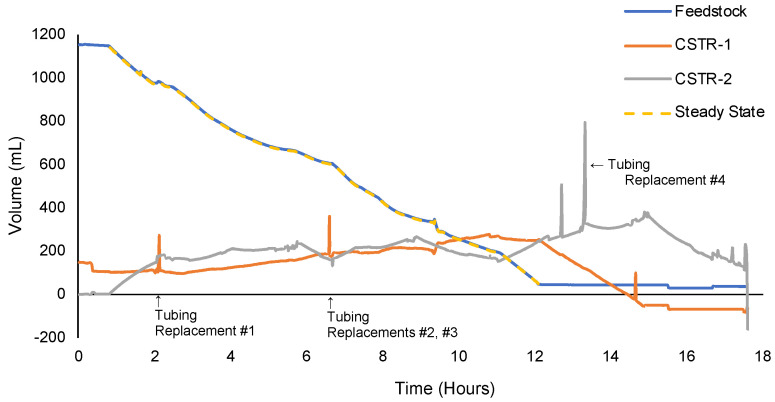
Process Monitoring Data from Run 2.

In run 2, a larger-scale continuous manufacturing experiment, WS-2 and WS-3 were employed to approximate fill levels in the CSTRs in addition to WS-1 measuring the feedstock. The process monitoring results are shown in Figure 9. Although both the PTFE and the C-Flex tubing were rated as compatible with ClSO_3_H, the heated reaction mixture slowly degraded the C-Flex tubing. More issues arose with C-Flex tubing compatibility as the process scaled. Any use of C-Flex tubing resulted in unsafe operating conditions and is explicitly not recommended. The miscalibration of P-1 and tubing degradation of the C-Flex portions of P-2 and P-3 led to high inconsistency. Although P-2 and P-3 were manually adjusted to maintain the set masses of CSTR-1 and CSTR-2, many tubing replacements shifted equipment on the scales, compromising the accuracy of scale readings. Approximate volumetric averages of 174.5 and 187.8 mL for CSTR-1 and CSTR-2, respectively, were far from the 150 mL volumetric setpoint. Miscalibration of P-1 and the cascading effect on P-2 and P-3 led to significantly reduced throughput and increased waste as P-4 and P-5 remained fairly accurate. The lack of process control and C-Flex tubing led to large residence time distributions. The ‘state steady’ trendline is applied where the starting material (P-1) is actively flowing through the system after the start-up period, although operationally a true dynamic equilibrium was never achieved.

**Figure 10:**
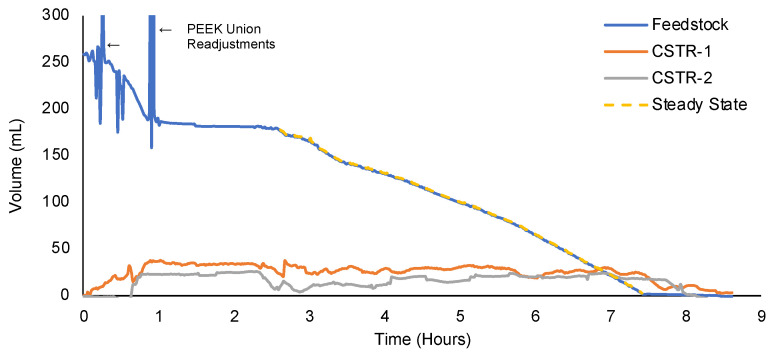
Process Monitoring Data from Run 3.

The experimental configuration using PTFE-compatible peristaltic pump heads and the total elimination of C-flex tubing for Run 3 is shown in Figure S12. Run 3 was used to validate the effectiveness of increasing from 6 to 8 eq. ClSO_3_H in flow and to assess the viability of the PTFE pump heads for a final large-scale manufacturing run. The PTFE tube sets were highly effective at transferring the heated crude reaction mixture, and no tubing replacements were required (Figure 10). The flowrate of feedstock with PTFE pump heads in Run 3 was consistent, as demonstrated by the relatively smooth decline. Although enabling more resistance to corrosion, the PTFE pump heads had significant issues with calibration. The digital reading of the flowrate from the pump display was not accurately signaling the actual feedstock flowrate. The stock flowrate was adjusted manually based on real-time scale data towards the target flowrate. Even with manual adjustments, the actual stock flowrate (0.578 mL/min) differed from the actual target flowrate (0.67 mL/min). The relative levels of CSTR-1 and CSTR-2 were also far from the desired 40 mL setpoint, averaging 26.7 and 17.6 mL with standard deviations of 3.2 and 4.8 mL, respectively. The high inconsistency and deviation from the setpoint can be attributed to the difficulty of manually adjusting the flowrates. The lack of process setpoint reliability and control established a clear motivation for an automated process. 

**Figure 11:**
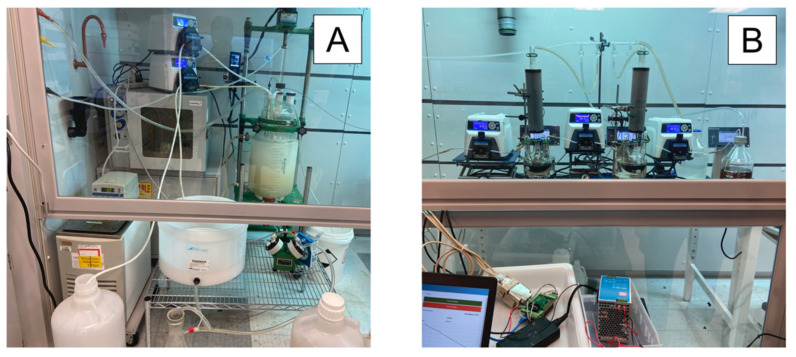
Walk-in Fume Hood Setup for Run 4. (**A**) Continuous isolation; and (**B**) reaction and process control systems.

**Figure 12:**
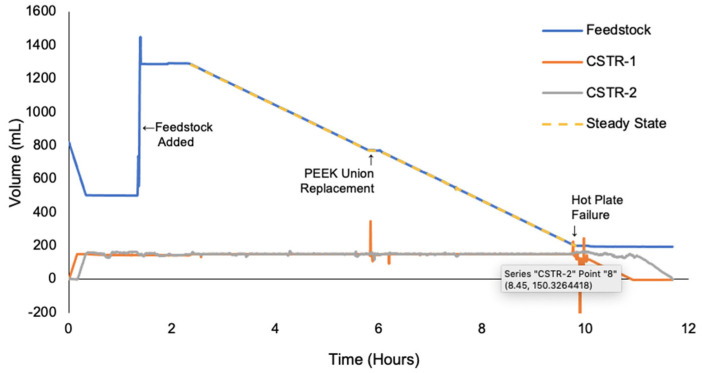
Process Monitoring Data from Run 4.

Automated pumping controls for the startup, steady state, and shutdown procedures were implemented based on the real-time data from the weighing scales. In Run 4, the PID controllers were tested in a large-scale synthesis. The setup for the final continuous manufacturing Run 4 is shown in Figure 11, and the process monitoring data are shown in Figure 12. The PID controller was highly effective in controlling residence time and flowrates. During the 12 h experiment, the first notable equipment failure was the PEEK union from the PTFE pump head to the PTFE tubing melting, which required replacement. Secondly, the hotplate under CSTR-1 produced an error message that halted heating and required removal to fix. It was not possible to readjust the hot plate without effecting the calibration of WS-2; therefore, P-1 was halted with about 54 g of starting material remaining in the stock. The material in CSTR-1 was still processed and collected. 

The process monitoring results are summarized in Table 7 and correspond to the plotted data in Figure S11, Figure 9, Figure 10 and Figure 12. Clearly, the most significant challenge faced during the development of a continuous chlorosulfonation method was the accurate and consistent transfer of heated ClSO_3_H. The process monitoring data revealed that the degradation of transfer tubing resulted in unstable flowrates, skewed residence times, and reduced throughput for initial runs. These issues were mitigated by the application of peristaltic PTFE tubing and the implementation of process controllers. The implementation of weighing scales proved to be an effective strategy for process monitoring. 

The analytical results for the product of each continuous manufacturing run are shown in Table 8. An improvement in yield is shown for Run 3 and Run 4 compared to Run 1 and Run 2, respectively. The total time to yield the product was reduced significantly by process automation in Run 4. Residual diglyme was found by ^1^H NMR to be present for Run 3 and Run 4. The amount of water required for the final wash to remove the residual diglyme did not scale up directly from the batch experiment (Table 5), and its presence lowered the HPLC mass assay of the product. Further investigation is needed to increase the purity of the large-scale diglyme co-solvent precipitation method to that of its batch counterpart. The highest equivalent of water only (62 vol.), used in Run 2, appears to be the most scalable method for the highest purity isolation despite the large amount of waste generated.

For both chemistry methods, the yield was higher during the smaller-scale synthesis runs compared to their following scale-ups. In Run 4, the area percent of sulfonic acid was much higher than usual, also due to insufficient washing. The amount of water for the isolation was reduced from 62 eq. (Run 2) to 9 eq. (Run 4). Throughout all the continuous manufacturing runs, the increased yield and throughput can be attributed to improved process control and chemistry optimization. 

**Figure 13:**
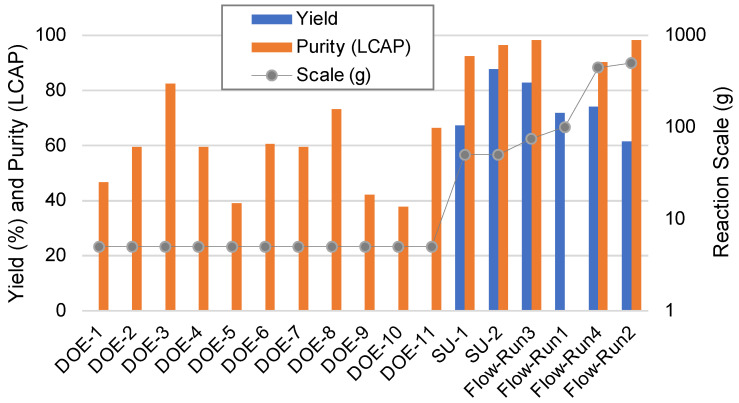
Summary of Yield and Purity for all Reaction Scales.

Several scales of batch and flow chemistry reactions were conducted throughout the course of this work. A pictorial graph that summarizes the available yields, purities (LCAP), and scale of each reaction is shown in Figure 13. Although the total yield generally decreased as the process was scaled, the spacetime yield of the continuous process improved significantly compared to the optimized batch conditions. For example, the optimized batch process SU-2 produced approximately 65 g in 6.5 h. The final flow chemistry experiment (Run 4) produced 500 g in 12 h with only twice the volume of heated chlorosulfonic acid. Accordingly, the spacetime yields for the best batch and flow chemistry processes were 0.072 and 0.139 g mL^−1^ h^−1^, respectively. 

## 4. Conclusions

The application of continuous manufacturing for a pharmaceutically relevant chlorosulfonation reaction provided several key advantages compared to the batch process. The spacetime yield of the final flow chemistry experiment was nearly doubled from the optimized scaled-up batch procedure. The continuous method also facilitated the controlled release and entrapment of toxic gaseous byproducts when compared to its batch counterpart. The development of an automated process control scheme greatly reduced operator exposure and the likelihood of human error. The effectiveness of the automation scheme and the reduction in maloperations were demonstrated by gravimetric process monitoring data. The PID controllers were created with open-source software and can generally maintain steady-state operation for any series of CSTRs that utilize gravimetric readings from weighing scales to approximate fill levels. This control strategy is recommended for reaction systems that have low compatibility with containment materials. Overall, this work demonstrates the utility of in-series CSTRs in the continuous manufacturing of a pharmaceutical intermediate and offers an alternative to a hazardous batch scale-up procedure. Future studies to evaluate the application of this process to high-impact, industrially relevant precursors are recommended. 

## Figures and Tables

**Table 1:** Drugs Containing Sulfonyl Functionalization.

Drug Name(s)	Function
Chlorothiazide/Hydrochlorothiazide	Diuretic/Antihypertensive [11]
Sildenafil Citrate	Phosphodiesterase Inhibitor [10,12]
Sulfamethoxazole	Chemotherapeutic [13]
Sulfasalazine	Anti-inflammatory [14]
Dorzolamide	Ocular Hypertension [15]
Tolbutamide	Anti-diabetic [16]
Celecoxib	Nonsteroidal Anti-inflammatory [17]

**Table 2:** DOE Model Factors and Responses.

Effective Factor	Abbreviation	Low Level (−1)	Medium Level (0)	High Level (+1)
Temperature	A	130	140	150
ClSO_3_H Equivalents	B	5	7.5	10
**Response**	**Abbreviation**		**Goal**	
Product (LCAP)	R1		Maximize	
Starting Material (LCAP)	R2		Minimize	
Sulfonic Acid (LCAP)	R3		In Range (Min–Max)	

**Table 3:** Experimental Design by Rotatable CCD for Two Factors of Temperature and Molar Equivalents of ClSO_3_H (Eq.).

DOE Batch Experiment	Temperature (°C)	Eq. (ClSO_3_H)
1	130	10
2	140	8
3	155	8
4	140	8
5	130	5
6	140	11
7	140	8
8	150	5
9	125	8
10	140	4
11	150	10

**Table 4:** Selected Multivariate Optimization Solution.

Temperature (°C)	Eq. ClSO_3_H	Product (LCAP)	Starting Material (LCAP)	Sulfonic Acid (LCAP)
155.1	7.3	87.4	1.0	16.5

**Table 5:** Scale-Up Batch Chemistry and Precipitation Conditions.

Scaled-Up Batch	Time (h)	Eq. ClSO_3_H	Temp. of Quench (°C)	Precipitation	Isolated Yield	Sulfonyl Chloride Mass Assay
SU-1	3	6.0	0	70 vol. ice H_2_O, 5 mL/min	67.3%	90.0%
SU-2	2	8.6	−5	8 vol. H_2_O, 0.8 vol. Diglyme, 2 mL/min	87.7%	92.0%

Reaction at reflux conditions (155 °C). No starting material was detected.

**Table 6:** Flow Process Parameters.

Run	Starting Material Processed (g)	Eq. ClSO_3_H	Precipitation Fluid Amount (mL/g Starting Material)	Tubing	WS-1 Monitoring	WS-2/3 Monitoring	P-1/2/3 Control
1	100	6	40 vol. H_2_O	C-Flex/ PTFE	Yes	No	Manual
2	500	6	62 vol. H_2_O	C-Flex/ PTFE	Yes	Yes	Manual
3	75	8	32 vol. H_2_O, 2.9 vol. Diglyme	PTFE	Yes	Yes	Manual
4	446	8	9 vol. H_2_O, 1 vol. Diglyme	PTFE	Yes	Yes	PID

**Table 7:** Process Monitoring Results.

Run	CSTR-1/2 Target (mL)	CSTR-1 Mean Volume (mL) +/− STDEV	CSTR-2 Mean Volume (mL) +/− STDEV	Feedstock Flowrate Target (mL/min)	Feedstock Flowrate Actual (mL/min)	R^2^
1	30	N/A	N/A	0.5	0.502	0.9952
2	150	174.5 +/− 57.0	187.8 +/− 46.1	2.5	1.499	0.9947
3	40	26.7 +/− 3.2	17.6 +/− 4.8	0.67	0.578	0.9925
4	150	150.1 +/− 3.3	149.7 +/− 2.9	2.5	2.4	0.9995

The coefficient of determination (R^2^) is shown for the linear fit of feedstock data during steady-state operation.

**Table 8:** Flow Process Chemistry Results.

Run	Time (Hours)	Yield **	Sulfonyl Chloride (LCAP)	Starting Material (LCAP)	Sulfonic Acid (LCAP)	Sulfonyl Chloride Mass Assay
1 *	11	71.9%	N/A	N/A	N/A	N/A
2	18	61.4%	98.2%	0.0%	1.8%	94.0%
3	8	82.8%	98.2%	1.1%	0.7%	86.9%
4	12	74.0%	90.2%	1.8%	6.8%	84.9%

* An accurate analytical method was yet to be determined during Run 1. ** Yield adjusted for product purity.

## Data Availability

The authors confirm that the data supporting the findings of this study are available within the article and its supplementary materials.

## Supplementary Materials

The following supporting information can be downloaded at: https://www.mdpi.com/article/10.3390/molecules28104213/s1, Figure S1. Chromatograph, HPLC Method 1: Run 2. Figure S2. Chromatograph, HPLC Method 2: Run 3. Figure S3. Kinetic Plot of DOE Batch Experiment 1. Figure S4. Kinetic Plot of DOE Batch Experiment 4. Figure S5. Kinetic Plot of DOE Batch Experiment 5. Figure S6. Kinetic Plot of DOE Batch Experiment 6. Figure S7. Kinetic Plot of DOE Batch Experiment 7. Figure S8. Kinetic Plot of DOE Batch Experiment 8. Figure S9. Kinetic Plot of DOE Batch Experiment 9. Figure S10. Kinetic Plot of DOE Batch Experiment 11. Figure S11. Process Monitoring Data from Run 1. Figure S12. Fume Hood Setup for Run 3. Table S1. Pumping Flowrates. Table S2. ANOVA of Product (R1). Table S3. ANOVA of Starting Material (R2). Table S4. ANOVA of Sulfonic Acid (R3).

## Sample Availability

Samples of the compounds are not available from the authors.

## References

[1] Aziz J., Hamze A. (2020). An Update on the Use of Sulfinate Derivatives as Versatile Coupling Partners in Organic Chemistry. Org. Biomol. Chem..

[2] Ruchika S.S., Kumar D., Gohri S. (2016). Therapeutic Aspects of Sulfonylureas: A Brief Review. J. Chem. Pharm. Res..

[3] Kołaczek A., Fusiarz I., Lawecka J., Branowska D. (2014). Biological Activity and Synthesis of Sulfonamide Derivatives: A Brief Review. Chemik.

[4] Alavi S., Mosslemin M.H., Mohebat R., Massah A.R. (2017). Green Synthesis of Novel Quinoxaline Sulfonamides with Antibacterial Activity. Res. Chem. Intermed..

[5] Irfan A., Ahmad S., Hussain S., Batool F., Riaz H., Zafar R., Kotwica-Mojzych K., Mojzych M. (2021). Recent Updates on the Synthesis of Bioactive Quinoxaline-Containing Sulfonamides. Appl. Sci..

[6] Ali A.T., Mosa M.N., Alshaheen Z.G., Muhammad-Ali M.A. (2020). Synthesis, Characterization and Antibacterial Evaluation of Oxoazetidin—Benzene Sulfonamide Derivatives as a Hybrid Antimicrobial Agents. Syst. Rev. Pharm..

[7] Ahjel S.W., Hassan S.M., Hussein S.F., Hadi N.R., Awad S.M. (2020). Antineoplastic Effect of New Synthesized Compounds of 2-Thiouracil Sulfonamide Derivatives against Ovarian and Breast Carcinoma Cells “In Vitro Study”. Syst. Rev. Pharm..

[8] WHO World Health Organization Model List of Essential Medicines 22nd List, 2021.

[9] Hernandez I., Gul Z., Gellad W.F., Davies B.J. (2021). Marked Increase in Sales of Erectile Dysfunction Medication During COVID-19. J. Gen. Intern. Med..

[10] Dunn P.J., Galvin S., Hettenbach K. (2004). The Development of an Environmentally Benign Synthesis of Sildenafil Citrate (Viagra^TM^) and Its Assessment by Green Chemistry Metrics. Green Chem..

[11] Gyűjtő I., Simig G., Porcs-Makkay M., Volk B. (2020). Synthesis and Chemistry of 1,2,3-Benzothiadiazine 1,1-Dioxide Derivatives: A Comprehensive Overview. Chemistry.

[12] Raghava Reddy A.V., Srinivas G., Takshinamoorthy C., Peruri B.G., Naidu A. (2016). A Facile, Improved Synthesis of Sildenafil and Its Analogues. Sci. Pharm..

[13] Abdelmoniem A.M., Abdelrahman M.G.M., Ghozlan S.A.S., Abdelhamid I.A. (2019). Synthesis of Novel Hexahydroquinolines and 6-Amino-2-Oxopyridine-3,5-Dicarbonitriles Incorporating Sulfamethoxazole via [3 + 3] Annulation. J. Heterocycl. Chem..

[14] Hofmann D., Gans E., Krüll J., Heinrich M.R. (2017). Sustainable Synthesis of Balsalazide and Sulfasalazine Based on Diazotization with Low Concentrations of Nitrogen Dioxide in Air. Chem. A Eur. J..

[15] Kouchak M., Mahmoodzadeh M., Farrahi F. (2019). Designing of a PH-Triggered Carbopol^®^/HPMC In Situ Gel for Ocular Delivery of Dorzolamide HCl: In Vitro, In Vivo, and Ex Vivo Evaluation. AAPS PharmSciTech.

[16] Hunter R., Msutu A., Dwyer C.L., Emslie N.D., Hunt R.C., Bezuidenhoudt B.C.B. (2011). Facile One-Pot Synthesis of Carbamoylbenzotriazoles Directly from CO Synthesis of Tolbutamide. Synlett.

[17] Habeeb A.G., Praveen Rao P.N., Knaus E.E. (2001). Design and Synthesis of Celecoxib and Rofecoxib Analogues as Selective Cyclooxygenase-2 (COX-2) Inhibitors: Replacement of Sulfonamide and Methylsulfonyl Pharmacophores by an Azido Bioisostere. J. Med. Chem..

[18] Saxena N., Srivastav N., Kumar A., Anjali A. (2022). Adoption of Green Methodology in Industry for the Synthesis of Sildenafil Citrate & Celecoxib: A Case Study. Mater. Today Proc..

[19] Alkan-Zambada M., Hu X. (2019). Cu-Catalyzed Photoredox Chlorosulfonation of Alkenes and Alkynes. J. Org. Chem..

[20] Ren H., Maloney K.M., Basu K., Di Maso M.J., Humphrey G.R., Peng F., Desmond R., Otte D.A.L., Alwedi E., Liu W. (2020). Development of a Green and Sustainable Manufacturing Process for Gefapixant Citrate (MK-7264) Part 1: Introduction and Process Overview. Org. Process Res. Dev..

[21] Zhang Z., Zhao Y., Geng H. (2020). Chlorosulfonation of Acetanilide in a Dual-Temperature-Zone Silicon Carbide Microchannel Reactor and Synthesis of Sulfasalazine. Chin. J. Org. Chem..

[22] Porta R., Benaglia M., Puglisi A. (2016). Flow Chemistry: Recent Developments in the Synthesis of Pharmaceutical Products. Org. Process Res. Dev..

[23] Baumann M., Moody T.S., Smyth M., Wharry S. (2020). A Perspective on Continuous Flow Chemistry in the Pharmaceutical Industry. Or.g Process. Res. Dev..

[24] Cole K.P., Johnson M.D. (2018). Continuous Flow Technology vs. the Batch-by-Batch Approach to Produce Pharmaceutical Compounds. Expert Rev. Clin. Pharmacol..

[25] Hernando M.V., Moore J.C., Howie R.A., Castledine R.A., Bourne S.L., Jenkins G.N., Licence P., Poliakoff M., George M.W. (2022). High Yielding Continuous-Flow Synthesis of Norketamine. Org. Process Res. Dev..

[26] Diab S., McQuade D.T., Gupton F.B., Gerogiorgis D.I. (2019). Process Design and Optimization for the Continuous Manufacturing of Nevirapine, an Active Pharmaceutical Ingredient for HIV Treatment. Org. Process Res. Dev..

[27] Armstrong C.T., Pritchard C.Q., Cook D.W., Ibrahim M., Desai B.K., Whitham P.J., Marquardt B.J., Chen Y., Zoueu J.T., Bortner M.J. (2019). Continuous Flow Synthesis of a Pharmaceutical Intermediate: A Computational Fluid Dynamics Approach. React. Chem. Eng..

[28] Sagmeister P., Lebl R., Castillo I., Rehrl J., Kruisz J., Sipek M., Horn M., Sacher S., Cantillo D., Williams J.D. (2021). Advanced Real-Time Process Analytics for Multistep Synthesis in Continuous Flow*. Angew Chem. Int. Ed. Engl..

[29] Vinet L., di Marco L., Kairouz V., Charette A.B. (2022). Process Intensive Synthesis of Propofol Enabled by Continuous Flow Chemistry. Org. Process Res. Dev..

[30] Cole K.P., Groh J.M., Johnson M.D., Burcham C.L., Campbell B.M., Diseroad W.D., Heller M.R., Howell J.R., Kallman N.J., Koenig T.M. (2017). Kilogram-Scale Prexasertib Monolactate Monohydrate Synthesis under Continuous-Flow CGMP Conditions. Science.

[31] Armstrong C., Miyai Y., Formosa A., Thomas D., Chen E., Hart T., Schultz V., Desai B.K., Cai A.Y., Almasy A. (2021). On-Demand Continuous Manufacturing of Ciprofloxacin in Portable Plug-and-Play Factories: Development of a Highly Efficient Synthesis for Ciprofloxacin. Org. Process Res. Dev..

[32] Jolliffe H.G., Gerogiorgis D.I. (2016). Plantwide Design and Economic Evaluation of Two Continuous Pharmaceutical Manufacturing (CPM) Cases: Ibuprofen and Artemisinin. Comput. Chem. Eng..

[33] Yazdanpanah N., Cruz C.N., O’Connor T.F. (2019). Multiscale Modeling of a Tubular Reactor for Flow Chemistry and Continuous Manufacturing. Comput. Chem. Eng..

[34] Glace M., Wu W., Kraus H., Acevedo D., Roper T.D., Mohammad A. (2023). The Development of a Continuous Synthesis for Carbamazepine Using Validated In-Line Raman Spectroscopy and Kinetic Modelling for Disturbance Simulation. React. Chem. Eng..

[35] Armstrong C., Miyai Y., Formosa A., Kaushik P., Rogers L., Roper T.D. (2023). Leveraging First-Principles and Empirical Models for Disturbance Detection in Continuous Pharmaceutical Syntheses. J. Flow Chem..

[36] Miyai Y., Formosa A., Armstrong C.T., Marquardt B., Rogers L., Roper T.D. (2021). PAT Implementation on a Mobile Continuous Pharmaceutical Manufacturing System: Real-Time Process Monitoring with In-Line FTIR and Raman Spectroscopy. Org. Process Res. Dev..

[37] Lee S.L., O’Connor T.F., Yang X., Cruz C.N., Chatterjee S., Madurawe R.D., Moore C.M.V., Yu L.X., Woodcock J. (2015). Modernizing Pharmaceutical Manufacturing: From Batch to Continuous Production. J. Pharm. Innov..

[38] ICH (2021). Continuous Manufacturing of Drug Substances and Drug Products (Q13).

[39] Polterauer D., Roberge D.M., Hanselmann P., Littich R., Hone C.A., Kappe C.O. (2022). A Continuous Flow Investigation of Sulfonyl Chloride Synthesis Using N-Chloroamides: Optimization, Kinetics and Mechanism. React. Chem. Eng..

[40] Malet-Sanz L., Madrzak J., Ley S.V., Baxendale I.R. (2010). Preparation of Arylsulfonyl Chlorides by Chlorosulfonylation of in Situ Generated Diazonium Salts Using a Continuous Flow Reactor. Org. Biomol. Chem..

[41] Cremlyn R.J. (2002). Chlorosulfonic Acid: A Versatile Reagent.

[42] Ignatovich Z.V., Ermolinskaya A.L., Ol’khovik V.K., Matveenko Y.V., Koroleva E.V. (2019). Synthesis of 10,10-Dioxo-10H-10λ6-Phenoxathiine-2,8-Dicarboxamides. Russ. J. Org. Chem..

[43] Chen Y., Sun X., Shan J., Tang C., Hu R., Shen T., Qiao H., Li M., Zhuang W., Zhu C. (2020). Flow Synthesis, Characterization, Anticoagulant Activity of Xylan Sulfate from Sugarcane Bagasse. Int. J. Biol. Macromol..

[44] Weissman S.A., Anderson N.G. (2014). Design of Experiments (DoE) and Process Optimization. A Review of Recent Publications. Org. Process Res. Dev..

[45] Politis S.N., Colombo P., Colombo G., Rekkas D.M. (2017). Design of Experiments (DoE) in Pharmaceutical Development. Drug Dev. Ind. Pharm..

[46] Liu H., Ricart B., Stanton C., Smith-Goettler B., Verdi L., O’Connor T., Lee S., Yoon S. (2019). Design Space Determination and Process Optimization in At-Scale Continuous Twin Screw Wet Granulation. Comput. Chem. Eng..

[47] Dong Y., Georgakis C., Mustakis J., Han L., McMullen J.P. (2020). Optimization of Pharmaceutical Reactions Using the Dynamic Response Surface Methodology. Comput. Chem. Eng..

[48] Node-RED (2022). Low-Code Programming for Event-Driven Applications.

[49] Node-Red (2021). Contrib-Pid: PID Loop Controller for Node-RED.

[50] Constable D.J.C., Curzons A.D., Cunningham V.L. (2002). Metrics to “green” Chemistry—Which Are the Best?. Green Chem..

