# Supplementary material for: An Automated Continuous Synthesis and Isolation for the Scalable Production of Aryl Sulfonyl Chlorides
Source: Molecules. 2023 May 20;28(10):4213. doi: 10.3390/molecules28104213 (PMC10223716; doi:10.3390/molecules28104213)
Supplement: Supplementary file 1 [file molecules-28-04213-s001.zip › molecules-2390564-supplementary.pdf]

5. Supplementary Information

5.1 Analytical Information

5.1.1 HPLC Method 1 Details: Run 2 and Batch Experiments

Column: Agilent Eclipse XDB-C18 (4.6 x 250 mm; 5 µm)
Mobile Phase A: 0.1% Phosphoric acid in Water (1 mL HPLC-grade H3PO4 in 1000 mL HPLC-grade water)
Mobile Phase B: Acetonitrile
Injection volume: 1 µL
Column temp.: 30 °C
Flowrate: 1.5 mL/min isocratic at 40% B for 12 min
Detector wavelength(s): 255 nm (primary), 210 nm (for information only)

Retention time of Sulfonyl Chloride: ~9.6 min
Retention time of Sulfonic Acid: ~1.5 min

5.1.2 HPLC Method 2 Details: Runs 3 and 4

Column: Agilent Eclipse XDB-C18 (4.6 x 250 mm; 5 µm)
Mobile Phase A: 0.1% Phosphoric acid in Water (1 mL HPLC-grade H_3PO_4 in 1000 mL HPLC-grade water)
Mobile Phase B: Methanol
Gradient: 25% Methanol increased linearly to 75 % over 15 min; hold at 75% for 5 min; 4 min post time
Injection volume: 1 µL
Column temp.: 30 °C
Flowrate: 1.5 mL/min
Detector wavelength(s): 235 nm (primary), 275, 210 nm (for information only)

Retention time of Sulfonyl Chloride: 15.8 min
Retention time of Starting Material: 14.7 min
Retention time of Sulfonic Acid: 4.5 min

5.1.3 Number of HPLC Mass Assay Sampling Procedures

Run 2–10 Samples (Average)
Run 3–1 Sample
Run 4–3 Samples (Mass-weighted average)

5.2 Supporting Figures and Tables

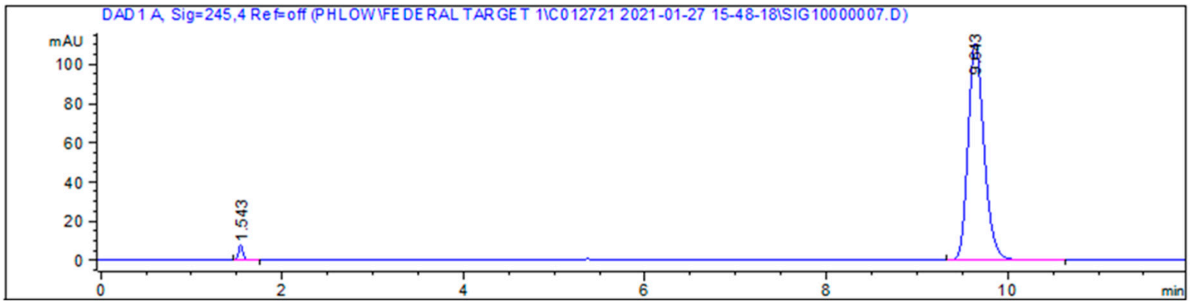

Figure S1. Chromatograph, HPLC Method 1: Run 2

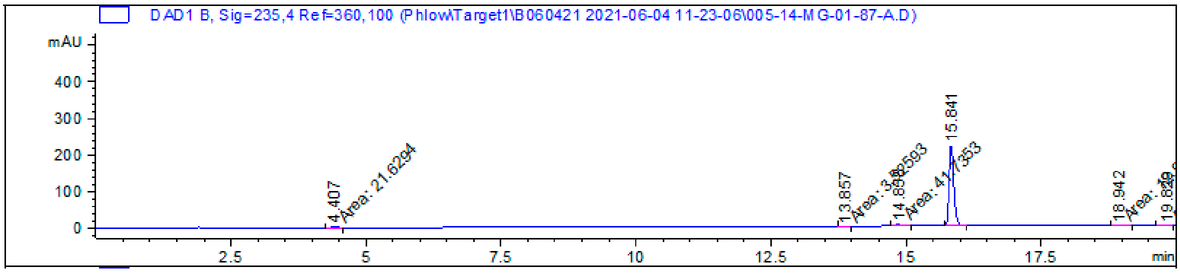

Figure S2. Chromatograph, HPLC Method 2: Run 3

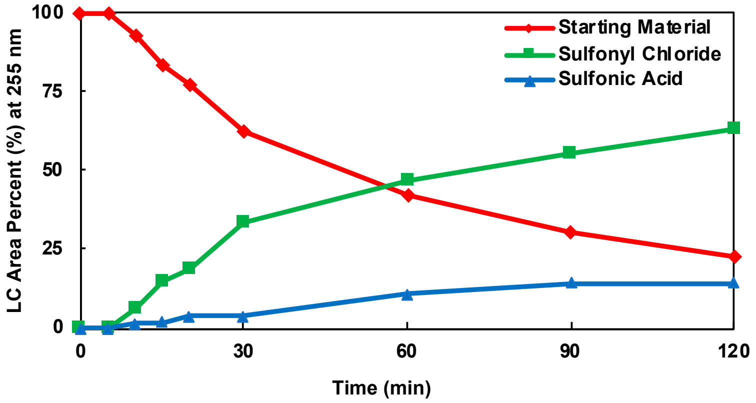

Figure S3. Kinetic Plot of DOE Batch Experiment 1

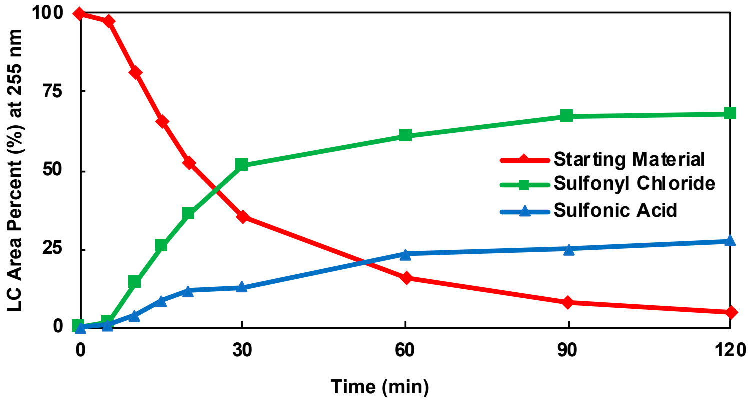

Figure S4. Kinetic Plot of DOE Batch Experiment 4

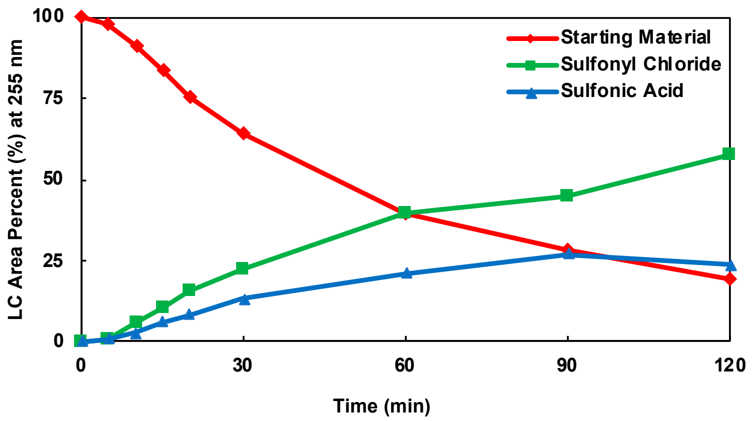

Figure S5. Kinetic Plot of DOE Batch Experiment 5

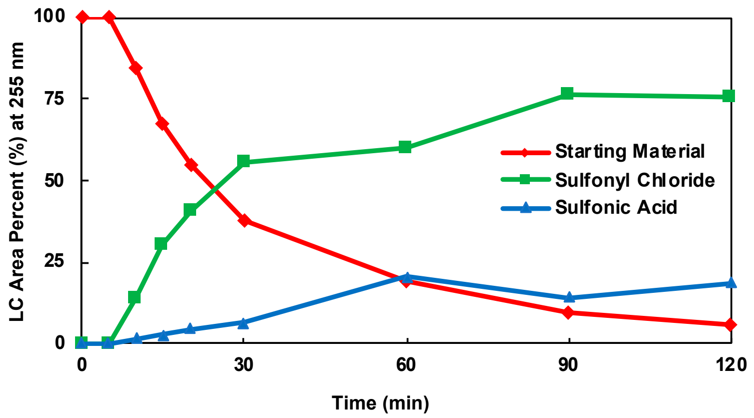

Figure S6. Kinetic Plot of DOE Batch Experiment 6

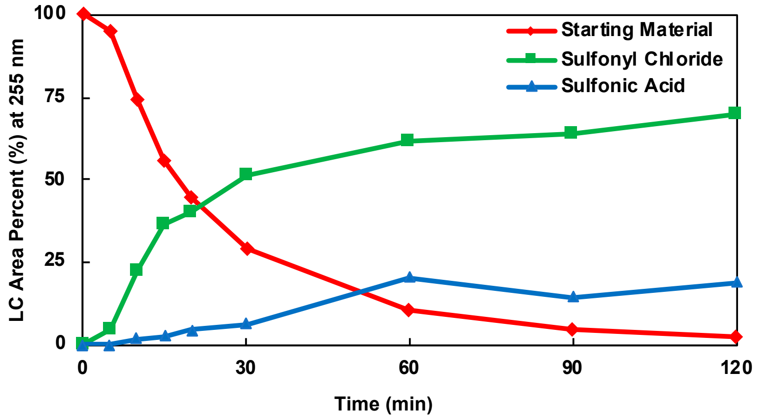

Figure S7. Kinetic Plot of DOE Batch Experiment 7

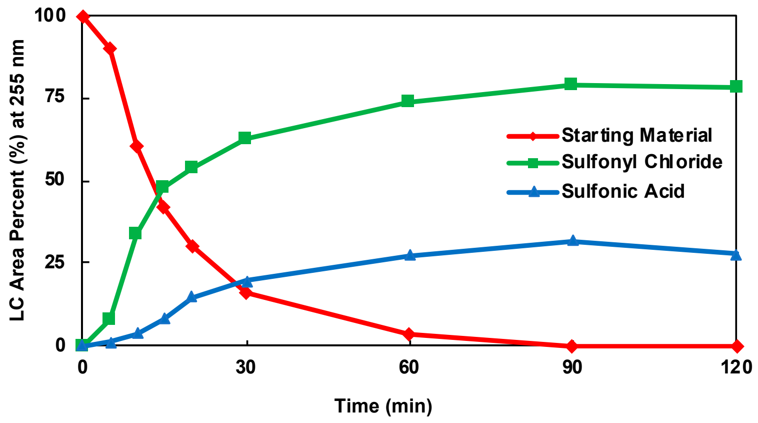

Figure S8. Kinetic Plot of DOE Batch Experiment 8

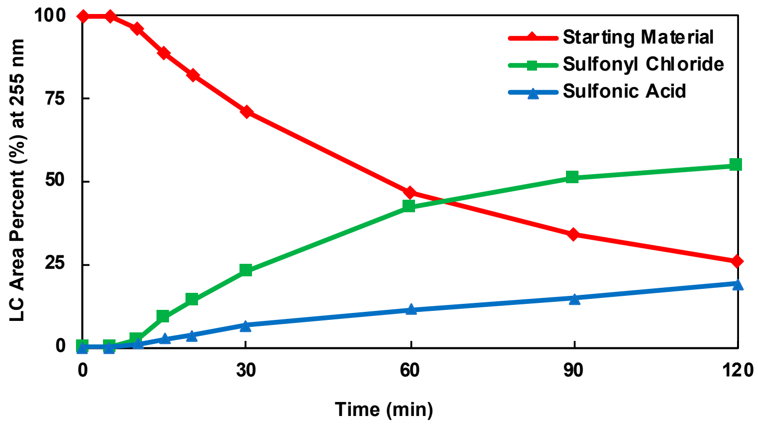

Figure S9. Kinetic Plot of DOE Batch Experiment 9

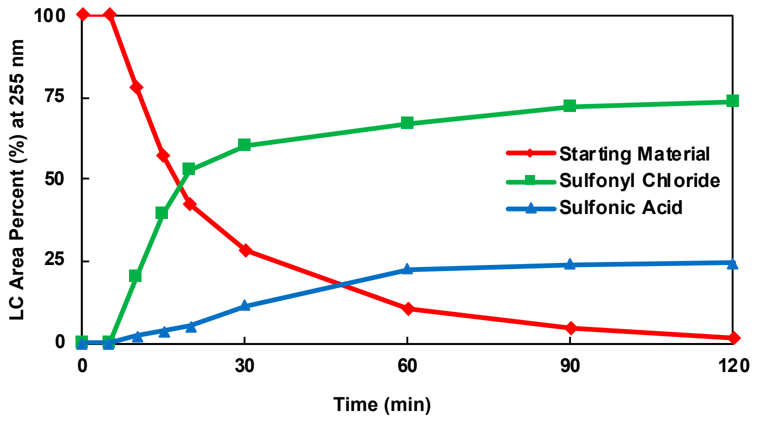

Figure S10. Kinetic Plot of DOE Batch Experiment 11

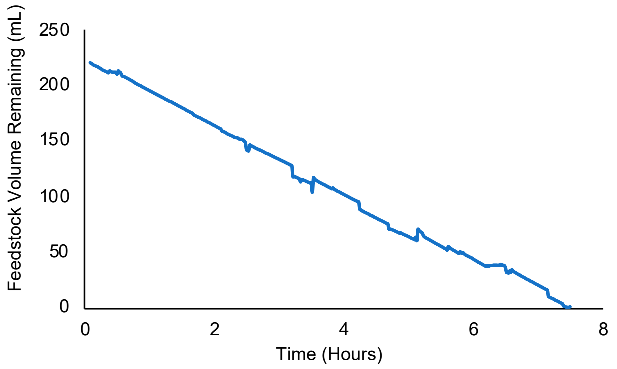

Figure S11. Process Monitoring Data from Run 1

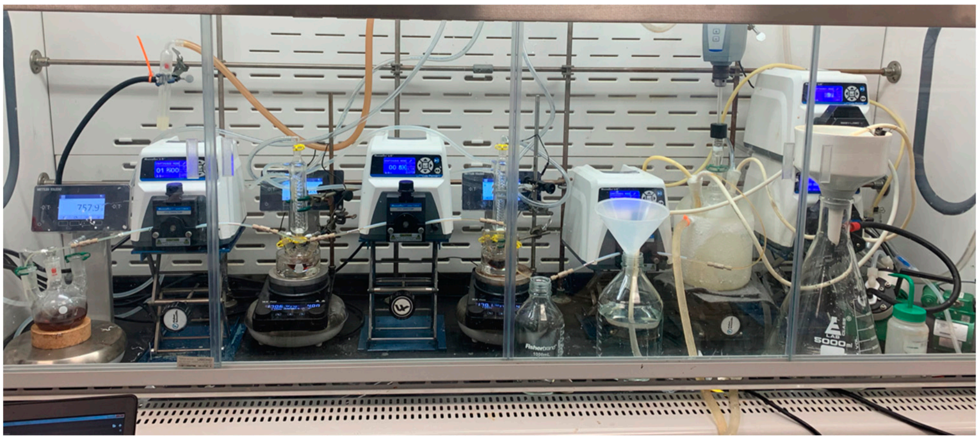

Figure S12. Fume Hood Setup for Run 3. PTFE pump heads and PTFE tube sets were purchased and implemented without the use of C-Flex tubing.

Table S1. Pumping Flowrates

Table	ISO-CSTR Setpoint (mL)	Jacket Set-point	P-1/P-2/P-3 (mL/min)	P-4 (mL/min)	P-5 On/Off (min:sec)	P-5 On (mL/min)
1	400	-5°C	0.5	12.5	0:25/2:35	98.3 mL/min
2	10000	-5°C	2.5	100	1:00/3:00	410 mL/min
3	500	-10°C	0.67	6.7	0:30/4:30	73.7 mL/min
4	7500	-5°C	2.5	7	0:10/1:02	200 mL/min

Table S2. ANOVA of Product (R1)

Source	Sum of Squares	df	Mean Square	F-value	p-value
Model	2057.35	7	293.91	41.34	0.0056
A-Temp.	803.59	1	803.59	113.03	0.0018
B-Eq.	260.53	1	260.53	36.65	0.009
AB	53.06	1	53.06	7.46	0.0718
A^2	49.87	1	49.87	7.01	0.0771
B^2	104.93	1	104.93	14.76	0.0311
A^2B	126.97	1	126.97	17.86	0.0242
AB^2	1.14	1	1.14	0.1605	0.7155
Residual	21.33	3	7.11		

Lack of Fit	0.7919	1	0.7919	0.0771	0.8073
Pure Error	20.54	2	10.27		
Cor. Total	2078.68	10			

Table S3. ANOVA of Starting Material (R2)

**Source**	**Sum of Squares**	**df**	**Mean Square**	**F-value**	**p-value**
Model	2397.11	5	479.42	29.53	0.001
A-Temp.	2185.97	1	2185.97	134.64	< 0.0001
B-Eq.	37.62	1	37.62	2.32	0.1884
AB	8.56	1	8.56	0.5274	0.5003
A^2	158.7	1	158.7	9.78	0.0261
B^2	20.88	1	20.88	1.29	0.3082
Residual	81.18	5	16.24		
Lack of Fit	47.54	3	15.85	0.942	0.5519
Pure Error	33.64	2	16.82		
Cor. Total	2478.29	10			

Table S4. ANOVA of Sulfonic Acid (R3)

**Source**	**Sum of Squares**	**df**	**Mean Square**	**F-value**	**p-value**
Model	0.0046	5	0.0009	26.38	0.0013
A-Temp.	0.0008	1	0.0008	24.18	0.0044
B-Eq.	0.0009	1	0.0009	26.6	0.0036
AB	0.0004	1	0.0004	12.48	0.0167
A^2	0.0019	1	0.0019	54.51	0.0007
B^2	3.93E-09	1	3.93E-09	0.0001	0.992
Residual	0.0002	5	0		
Lack of Fit	0.0002	3	0.0001	5.53	0.1571
Pure Error	0	2	9.45E-06		
Cor. Total	0.0048	10			
